# VDAC3 As a Potential Marker of Mitochondrial Status Is Involved in Cancer and Pathology

**DOI:** 10.3389/fonc.2016.00264

**Published:** 2016-12-23

**Authors:** Simona Reina, Francesca Guarino, Andrea Magrì, Vito De Pinto

**Affiliations:** ^1^Department of Biomedical and Biotechnological Sciences, University of Catania, Catania, Italy; ^2^National Institute of Biostructures and Biosystems (INBB), Rome, Italy

**Keywords:** VDAC3, mitochondria quality control, cancer, cysteine over-oxidation, aging, mitophagy, mitochondrial dysfunction

## Abstract

VDAC3 is the least known isoform of the mammalian voltage-dependent anion selective channels of the outer mitochondrial membrane. It has been recently shown that cysteine residues of VDAC3 are found over-oxidized. The VDAC3 cysteine over-oxidation was associated with the oxidizing environment and the abundance of reactive oxygen species (ROS) in the intermembrane space. In this work, we have examined the role of VDAC3 in general pathogenic mechanisms at the basis of mitochondrial dysfunction and involving the mitochondrial quality control. Many of the diseases reported here, including cancer and viral infections, are often associated with significant changes in the intracellular redox state. In this sense, VDAC3 bearing oxidative modifications could become marker of the oxidative load in the mitochondria and part of the ROS signaling pathway.

## Introduction

The outer mitochondrial membrane (OMM) is a continuous membrane that separates the cytosol from the inside of the organelle. It has long been imagined as a simple envelope, perforated by plenty of water-filled holes, as it has recently been illustrated in evocative AFM images (1). Nevertheless, the OMM can be considered the border of the mitochondria and any message to be communicated outside the mitochondrion has to be dealt with it. Furthermore, the OMM is involved in fusion–fission, communicates to the cell about the derangements in the bioenergetics processes, and rules the interaction of cytosolic molecules with the mitochondrion: these large or hydrophobic molecules require a specific docking structure. Cytoskeletal components (2, 3), ribosomes (4), and enzymes (5–8) contact the outer surface of mitochondria, or anchor to it, for structural or organizational purposes. The importance of OMM is demonstrated by the catastrophic results of its destruction: when the intactness of the OMM is destroyed, in the so-called permeability transition, the apoptotic cascade begins (9).

The most abundant protein located in the OMM is the family of pore structures called VDACs (for voltage-dependent anion selective channels). They are considered rather unspecific, hollow, water-filled pores (10–12): in contrast with Tom40, another pore-forming protein of the OMM, with similar structure, is devoted to channeling polypeptides to be folded in mitochondria (13). The apparent unspecific function contrasts with many roles, many issues, and many pathologies in which VDACs have been involved. Several important advancements derived by casual bumping of investigations into VDACs: once for all, the very first sequence, at protein level, of human VDAC1 was performed on a protein believed to be something else, a component of the immune system (14). There is thus a need for a deeper study of VDACs, addressing the more profound functions of this group of protein. In this work, we have focused our attention onto the least investigated mammalian VDAC isoform, named VDAC3, since it was the last one to be discovered (15). We report here a survey of its known feature and the information about its role in pathology. In general, however, a defective VDAC should not impact on a single pathology but instead be part of the mitochondrial dysfunction, in turn associated with a myriad of cellular pathological states.

## VDAC3 Structure: The Essential Information

The VDACs are a small family of proteins whose primary role is to form an aqueous pore through the OMM that allows the exchange of metabolites and molecules (10–12). In chordates, and in particular in mammals, three distinct VDAC isoforms are coded in the nucleus and targeted to the mitochondria (16). The VDAC sequences are conserved, have a length of about 280 amino acids, and, despite they form a hydrophilic barrel, they behave as integral membrane proteins in the isolation procedures, comparable to the transmembrane carriers (17, 18).

3D structures of mouse and human VDAC1 isoform have been experimentally determined by X-ray crystallography and NMR (19–21). Since the sequences are conserved, a similar structure has been hypothesized for the other two isoforms (22, 23). The VDAC2 structure from zebrafish has been solved, and it is very similar to the VDAC1 (24). Interestingly, the zebrafish VDAC2 has no N-terminal extension typical of this isoform in mammalian and has only one cysteine in its sequence (Cys 127). The β-strands are arranged in an antiparallel bunch strengthened by hydrogen bridges between each couple. Since the number of strands is odd, the first and last strands run in parallel: this is a peculiarity of mitochondrial VDACs in comparison with bacterial porins, which form an even, completely antiparallel β-barrel (25). The β-strands are connected by short turns or loops. As it is more evident for the bacterial porins (25), shorter turns crowd one side of the barrel [toward the intermembrane space (IMS)], and slightly longer loops the other side (the cytosol). The assignment of the turns and loops to a definite side of the membrane was a very important achievement (26) because it allows to investigate the availability of single amino acid residues to one of the two sides of the outer membrane. The last peculiarity of the VDAC structure is the N-terminal tail, a sequence of 25–36 amino acids (36 in VDAC2) predicted to form an exotic α-helix in a whole β-strands structure (27). The sequence has been assigned by structural determinations inside the pore, with a strong tendency to fold as an amphipathic α-helix. However, the exact arrangement of this segment requires further refinement, because there are slight differences in the available structures (28). The importance of the N-terminal tail has been highlighted in many works, since its involvement in the dynamic gating of the pore is possible.

The VDAC3 structure has not yet been obtained. Several bioinformatic predictions have been proposed, based on the large sequence similarity, with a β-barrel core almost identical to the other VDAC isoforms (23). Unfortunately, very little information about this isoform is present in the literature (29).

The puzzling question refers to the presence in the mitochondria of three different isoforms with very similar structure. The three VDACs are encoded by distinct genes located on different chromosomes (22, 30): they share the same exon–intron organization indicating that they are the result of not evolutionary far duplications (22, 30, 31): but the presence, the different expression levels (32–34), the different results of each gene K.O. (35–38) indicate that during evolution three VDAC genes were fixed in the chordate genome, most likely because they improved the cell (or the mitochondria) fitness.

## Recent Investigations on the Mammal VDAC3: Over-Oxidation of Cysteines

**Figure 1:**
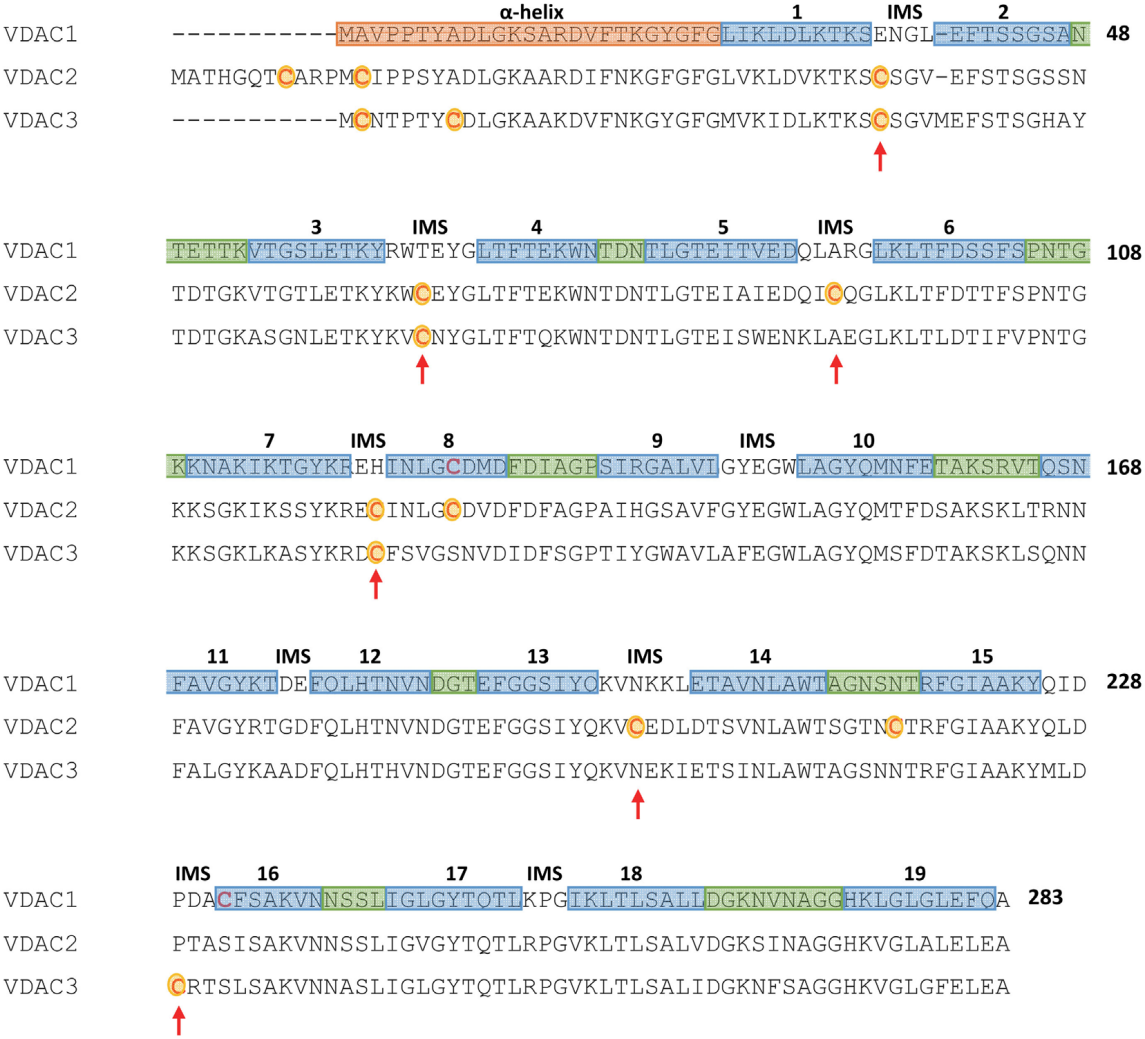
**Multi-alignment of human mammalian voltage-dependent anion selective channel (VDAC) isoforms**. The multi-alignment, obtained by the Clustal-X software, reports as a pink box the N-terminal sequence of VDAC1, in mauve the sequences forming the 19 β-strands, and in pale green the sequences corresponding to cytosol-exposed loops. The non-colored sequences correspond to the intermembrane-exposed turns (intermembrane space, IMS). The strands localization refers to the structure reported in Ref. (19), while the assignment of IMS or cytosolic loops follows the paper by Tomasello et al. (26). Cysteine residues of VDAC2 and VDAC3 are in red and outlined in yellow, and the red arrows show those cysteine residues protruding in the intermembrane space.

Our working hypothesis about a specific function of VDAC3 begun from the sequence analysis. It is evident that a striking difference among VDAC isoforms is the number of cysteines in the sequences. In human, VDAC1 has two Cys, VDAC2 has nine Cys, and VDAC3 has six Cys. Furthermore, it is indeed interesting that the three isoforms show different structural localization of their sulfur amino acids (Figure 1).

The sideness determination allowed the assignment of the cysteine residues position in interstrands loops or turns. It helped to speculate about their exposition to the water environment. While VDAC1 contains two Cys, located far from each other and interacting, respectively, with the water interior of the pore (Cys 232 in the β-strand 16) and with the hydrophobic, phospholipidic milieu (Cys 127 in the β-strand 8) (19, 39, 40), the other two isoforms’ cysteines mostly protrude toward the IMS (16, 26, 41). In particular, VDAC2 and VDAC3 show conserved cysteine residues at positions 36, 65, and 122; in addition, VDAC3 has Cys 229 exposed to the IMS, while VDAC2 has not only Cys 92 and 199 exposed to IMS but also Cys 127 in the middle of β-strand 8 and Cys 216 in the loop between β-strands 14 and 15. At the end, in VDAC2 and VDAC3, two cysteines are localized in the N-terminus moiety, thus in the water interior but only one of them is conserved at the same position in the two isoforms (Cys 2 in VDAC3 numbering) [the definition of the turns and loops is based on the human VDAC1 structure (19), thus it is predictive for the other isoforms; numbering follows the VDAC1 sequence, for the sake of clarity; please refer to the scheme in Figure 1]. Thus, human VDAC2 has more cysteines than VDAC3 but only in VDAC3 they look all preferentially exposed to the IMS.

The IMS is one of the oxidizing environments in the cell, together with the ER lumen (42). Its oxidizing power is a consequence of the proton unbalance across the inner mitochondrial membrane, due to the electron chain oxidation; the glutathione redox balance is also in favor of an oxidative potential (43, 44) and Complex III and Monoamine Oxidase directly pour reactive oxygen species (ROS) in the IMS (45, 46). It has been observed that VDACs are the conduit for the diffusion of the superoxide anion outside the organelle (47) and of hydroperoxide, produced by the dismutation of superoxide anion by SOD. Thus, we suspected that the VDAC cysteines exposed to the IMS could be a preferential target to discharge the oxidative load of the ROS in the compartment. We focused in particular on VDAC3 and, to get a molecular evidence of this hypothesis, we performed high resolution Mass Spectrometry analysis on VDAC3 derived from rat liver mitochondria (41). The results showed that (i) VDAC3 is electrophoretically heterogeneous (i.e., it is present with different mobility in SDS-PAGE) because the arrangement of oxidized cysteine is different in the various bands; (ii) different molecules contain different extents, in terms of type and quantity, of oxidized cysteine (we like to call them: “redox isomers”); (iii) the oxidation states of the cysteine are not random but some of them (Cysteines 2 and 8) are always found in a reduced (exactly derivatized by iodoacetamide) state, others (Cysteines 36, 65, and 229) in different oxidation levels (+1, +3, and +5); (iv) the oxidation to sulfinic (+3) or sulfonic (+5) acid is biologically irreversible. The only known reductase is thioredoxin/sulforedoxin system that targets sulfinic cysteine (48, 49), while there are no known enzymes able to reverse the sulfonic acid (+5) whose function is unknown and possibly related to either modify the protein electrostatic equilibrium or target it for degradation.

What can be the meaning of VDAC3 oxidations for the mitochondria within the cellular context?

## Relevance of Oxidation States of VDAC3 in Physiopathology: Is VDAC3 a Marker of the Mitochondrial Quality Control and Aging?

We have proposed, well before the discovery of over-oxidized cysteines, two explanations for the VDAC3 cysteines exceeding in number those present in VDAC1 (22). In the former hypothesis, the load of oxidations of VDAC3, whose role in the permeability of the OMM is not preeminent, can be considered as a defensive mechanism: the accumulation of damages on an apparently unimportant protein may drain the excess ROS. In the latter hypothesis, the accumulation of ROS modifications on the same protein, in addition to protect other molecules, can change the conformation and/or the docking capability of VDAC3 and this modification, in turn, signals the ROS load of the single mitochondrion to other structures within the cell.

**Figure 2:**
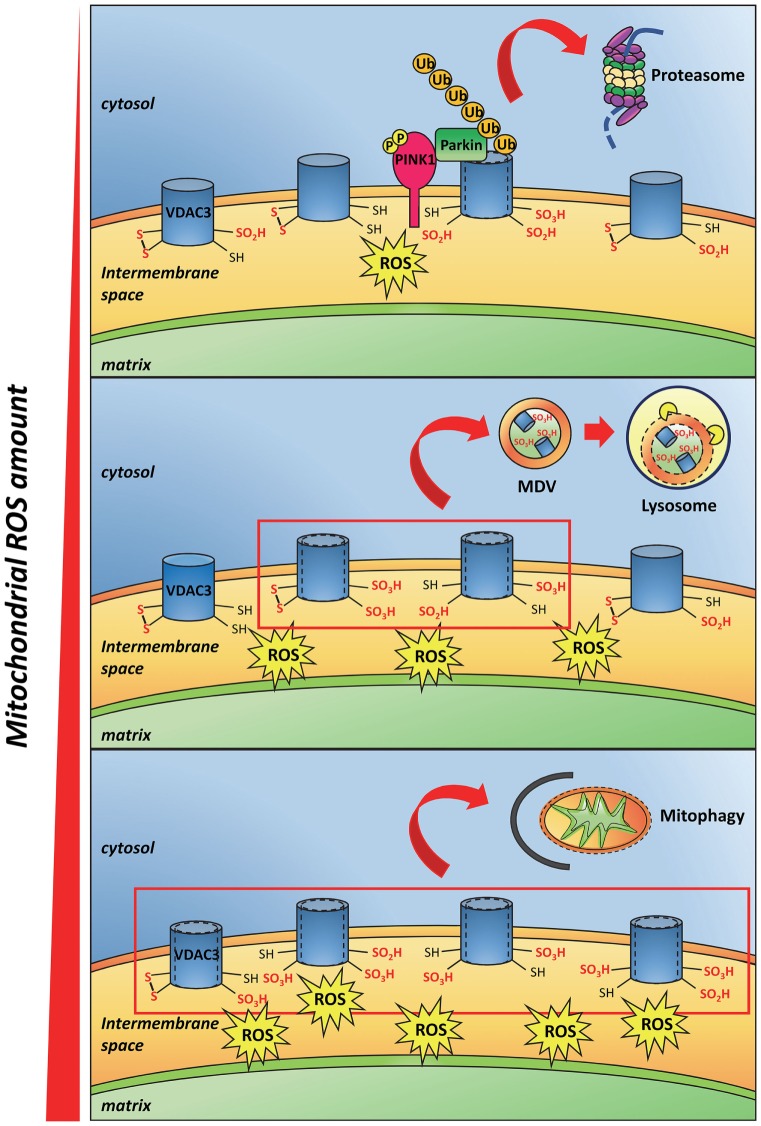
**Proposed model of mitochondrial reactive oxygen species (ROS) and VDAC3 cysteine residues interaction**. Little amounts of ROS oxidize some VDAC3 cysteines that protrude toward the mitochondrial intermembrane space up to sulfinic and sulfonic oxidation states. In addition to a conformational change in the protein, such irreversible modifications recruit the PINK/Parkin system that in turn ubiquitinates VDAC3. This step, followed by the proteasome degradation of the ubiquitinated protein, is preliminary to the mitochondrial quality control (*upper panel*). The progressive accumulation of ROS, due to mitochondrial stress, increases the amount of oxidized cysteines in VDAC3. This phenomenon stimulates the incorporation of single damaged proteins, or membrane patches containing damaged proteins, into mitochondria-derived vesicles, subsequently targeted to lysosomes (*middle panel*). When the ROS level reaches a maximum threshold, almost all VDAC3 proteins of the outer mitochondrial membrane become heavily modified by irreversible oxidations. Conformational changes derived from these modifications signal the redox state of the mitochondria to the rest of the cell. Damaged, reactive oxygen species-producing mitochondria are therefore removed through mitophagy (*lower panel*).

Recently, deletion of cysteines in engineered VDAC3 molecules (41, 50) suggested that the oxidized state of these residues can decrease the VDAC3 pore-conductance activity (41, 50, 51), even though the molecular mechanism of such hindrance to the conductance is not clear yet. Okazaki et al. proposed that the transient formation of a disulfide bridge inside the pore could strongly change the permeability (50). In our hands, we found that a different disulfide bridge can form but does not change much the permeation available diameter of the pore (41, 52). The modifications to the polypeptide chain conformation due to the cys oxidation can also affect the electrical charge disposition on the protein surface: in VDACs, the surfaces inside the barrel or at the mouths of the pore are hydrophilic (53). Furthermore, the insertion of negative charges due to sulfinic and sulfonic oxidation can provoke electric repulsions inside the chain or toward phospholipids, and next modify the conformation of the protein. A similar output has been evidenced in Ref. (52), where a molecular dynamic simulation has shown that, in case of disulfide formation between the closest cysteine residues available, the pore diameter would not change, while the exposition of residues, or of part of the N-terminal moiety, to the extra-membranous environment would change (52, 54). These conformational changes can be “sensed” in the cell as modification of the docking properties of the outer mitochondrial surface. The amounts of ROS in each mitochondrion can indeed cause VDAC cysteine oxidation proportional to the ROS concentration. We thus speculated that the quantitative extent of VDAC oxidative modifications on the mitochondrial surface can be used as a signal of oxidation load, allowing the monitoring of the ROS amounts in each mitochondrion or mitochondrial network section (Figure 2). At the end, oxidation of the cysteines in VDAC could provoke the binding or the release of yet unknown interactors.

VDAC2 is also rich in cysteines: it is possible that this isoform could also fulfill a similar function. The differences in number and location of cysteine residues do not support an overall overlapping of the VDAC2 and VDAC3 functions. Unfortunately, the molecular analysis of VDAC2 by mass spectrometry has not been completed, thus it is premature to draw conclusions about VDAC2.

It is well known that mitochondria are subjected to quality control systems that allow the cell to dispose malfunctioning organelles or just part of them. Such a quality control has the role to avoid that the cell is affected by a relevant mitochondrial dysfunction, an event reported in a large number of degenerative pathologies. The single affected organelle is targeted to lysosomes and destroyed. As an alternative, single proteins, or membrane patches are identified as damaged portions of the organelle and destroyed by a complex process involving the formation of mitochondria-derived vesicles (MDVs) and their targeting to lysosomes [reviewed in Ref. (55)].

The production of MDVs is a process stimulated by the mitochondrial stress. In a recent work where the MDV pathway has been dissected and characterized, it was demonstrated that MDVs are enriched in oxidized proteins derived from mitochondria and in particular from the outer membrane (56). The authors suggested indeed that the protein conformational changes subsequent to oxidation can initiate their incorporation into MDVs. The oxidized state of proteins was demonstrated with immunoblots targeting carbonylated amino acids (56). VDAC1 was found among the proteins in MDVs. Unfortunately, the oxidation state of cysteines was not investigated nor the presence of VDAC3 in MDVs.

It is peculiar for VDAC3 that the post-translational modification deletes the starting methionine in the sequence, leaving N-terminal residue the cysteine encoded as second amino acid (Cys 2). Cysteine at the N-terminal position is subjected to the so-called “N-end rule pathway,” a destructive process involving the oxidation of the N-terminal Cys, its selective arginylation and, at the end, its ubiquitination (53, 57). This pathway forwards the modified protein from the mitochondria through a retrotranslocation pathway to the cytosolic proteasome for its recycling, similar to the ER-associated degradation pathway (ERAD). In our studies, Cys 2 in VDAC3 was never found oxidized: the oxidized N-terminal cysteine is the precursor stage for the N-end pathway. Cysteine 2 in VDAC3 was instead always found involved in a disulfide bridge (41). During the MS pre-processing of the samples, Cys 2 was reduced and modified with iodoacetoamide. Iodoacetoamide irreversibly modifies the reduced cysteines, marking them as reduced or reducible in the conditions of analysis. The lack of any evidence of Cys 2 oxidation might just mean that each single protein altered in this way in the cell is immediately destroyed by the retrotranslocation pathway (N-end rule pathway). VDAC3 has been identified as a Parkin interactor and subjected to ubiquitination by Parkin in defective mitochondria (58). The PINK1/Parkin system is responsible for the elimination of defective mitochondria by autophagy (59). PINK1 is a kinase present, at very low levels, in the OMM, that is able to phosphorylate Parkin upon its recruitment from cytosol to the OMM. The phosphorylated Parkin starts ubiquitination of selected targets in the mitochondria and this event is considered preliminary to the mitochondrial quality control (or to mitophagy). Experiments by Sun et al. support a model in which VDACs are part of the machinery that recruits Parkin to the organelle (58). By mass spectrometry, VDAC isoforms have been found to be the most abundant Parkin-associated proteins, indicating their role as mitochondrial docking site for Parkin. Interestingly, while VDAC3 was found ubiquitinated, VDAC2 can bind Parkin but it was not detected ubiquitinated (58). This difference can be due to the lack of the N-terminal cysteine in the mature form of VDAC2. When the Cys 2 is reduced or forms a disulfide bridge (41, 50), the VDAC3 protein can follow the same degradation pathway followed by the other isoforms.

During aging, stem cells sustain damages until they become exhausted (60). To reduce and slow the accumulation of such damages, stem cell might segregate ruined subcellular components away from the new stem cell. This trick would be extremely useful, excluding damaged mitochondria from the new, daughter stem cells. Katajisto et al. (61) were able to monitor the fate of aged mitochondria by using a photoactivable GFP tag linked to an outer membrane protein, the Omp25. The conclusion of this study was that apportioning of mitochondria to daughter cells from a stem cell is not symmetric: old, aged mitochondria are confined to the somatic cell and not to the daughter stem cell (61). This interesting work showed that, in stem cell, a modification marking old mitochondria must exist, to permit the asymmetric apportioning that is useful to stem cells.

Can the over-oxidation of cysteines in VDAC3 be one of the markers of such aging? It is not straightforward to imagine that the cysteines, exposed to the IMS, can direct a signal on the other side of the membrane (cytosol). We hypothesize that the over-oxidation of cysteines and/or other modification-like formation or break of disulfide bridges (41, 50, 54) can modify the structure of the pore or the electrostatic properties of the water-exposed surfaces and the mobility of the N-terminus. This conformational change could be the real switch (see Figure 2 for a cartoon depicting this hypothesis). A further protein, whose interaction with VDAC3 is modified upon the pore conformational change, should thus be hypothesized as an intermediate messenger.

## Interactomic Analysis Confirms the Role of VDAC3 in Mitochondrial Protein Quality Control

The relationship of VDAC3 with cellular systems was highlighted in the catalog of proteins found to interact with this isoform *in vivo* by a TAP-Tag immunoprecipitation strategy and mass spectrometry identification (62). Proteins from the endoplasmic reticulum were found to be well represented: Grp75, Hsp70, GRP, and calreticulin. They are also markers of the MAM, the mitochondria-associated membranes, considered as contact point and exchange site between mitochondria and ER (63). Other crucial pathways were clearly correlated to VDAC3 based on the presence of interacting proteins involved in it. Proteins correlated to oxidative stress (GSTO-1, PRDX, and GSTK-1), proteins involved in the response to misfolded or unfolded proteins (YWHAQ, KCIP1, or SFN), proteasomal components and chaperons (PDI and Erp5) (64, 65), and proteins related to ribosome contact and control, were identified (62).

Isoforms of protein disulfide isomerases, peroxiredoxins, glutathione transferases, involved in maintaining the redox status, or glutathione *S*-transferase kinase 1 (GSTK-1) were among VDAC3 interactors (66, 67). Mammalian unfolded protein response utilizes two main mechanisms able to eliminate misfolded proteins: upregulation of chaperons and proteolytic degradation by ubiquitin–proteasome and autophagy–lysosome system (68). Proteins like the interesting but poorly known 14-3-3 protein theta, the protein kinase C-inhibitor, or the stratifin, are components of these pathways and were indeed among the interactors. Also, the ERAD (69, 70), where the endoplasmic reticulum faces accumulation of mis-folded or unfolded proteins, was found well represented with proteins like chaperones, oxidases, and thiol-isomerases (i.e., protein disulfide isomerases, calreticulin) (62).

A very interesting interactor of VDAC3 found in Ref. (62) is VCP (TER-ATPase). VCP is a member of the ERAD pathway and is involved in the “extraction” of protein from the OMM and other membranes to be directed to ubiquitination and degradation through ER, with or without stress conditions (64). VCP mutants have been correlated to the onset of some type of myopathies, of frontotemporal dementia and of Alzheimer disease, Parkinson’s disease, or amyotrophic lateral sclerosis, all pathologies where VDAC involvement has been already reported (71–74). VCP could be in charge of extracting the modified version of VDAC3, addressing it toward microtubules through cytoplasmic granules’ traffic (62) and enriching near the centrosome. VDAC3 has been detected at the centriole and its role in ciliogenesis has been proposed (75, 76). To draw a more careful and comparative comparison of the VDAC isoforms’ roles, the interactomic analysis of VDAC2, presently lacking, would be essential.

## VDAC in Pathologies

At dawning of VDAC research, when there were only suspects of more VDAC isoforms and the human genome sequence was not completed, it raised a substantial interest that the claim of VDAC1 lack in a human patient, suffering of an undiagnosed encephalomyopathy (77). The evidence come out from Western blot of the skeletal muscle biopsy of a 3-year-old child, suffering of dysmorphism, hypotonia, respiration and feeding problems, and seizures. The underlying idea was that suspected, but not diagnosed, mitochondrial pathologies could be due to the absence of a transport protein, located in the mitochondrial membranes. This strategy gave raise to a screening with anti-VDAC1 antibodies run on muscle mitochondria membranes. About 100 suspected patients were investigated for VDAC1 deficiency in the Netherlands and Italy (78). As reported in Ref. (71), the patients to be examined were selected on a biochemical basis, having to show (a) diminished substrate oxidation rates not caused by a disturbance in the respiratory chain, citric acid cycle, or pyruvate dehydrogenase complex or (b) normal activities of the respiratory chain enzymes and the pyruvate dehydrogenase complex, together with strong suspicion for mitochondrial disorder based on both clinical and clinical–chemical hallmarks (79). The search for VDAC deficiency at a protein level was nevertheless later abandoned because the paucity of putative patients and their spread. In addition, the lack of reliable antibodies, working specifically against each singular VDAC isoform, hindered the possibility to study different pores. Most of the information about VDACs and in particular VDAC3 involvement in pathologies stemmed, thus, by wide-scale analysis of gene expression or general proteomic surveys. In many of them, evidence of the presence of altered levels of VDAC isoforms and in particular of VDAC3 was often found (80, 81).

## Evidence of VDAC3 Involvement in Cancer

In a recent survey with the Ingenuity Pathway Analysis software on VDAC3 interacting proteins, aimed to discover the pathological processes underneath this group of proteins, it was noticed that the processes with the highest score, by far highest than the threshold, were cancer and reproductive system diseases (62).

**Figure 3:**
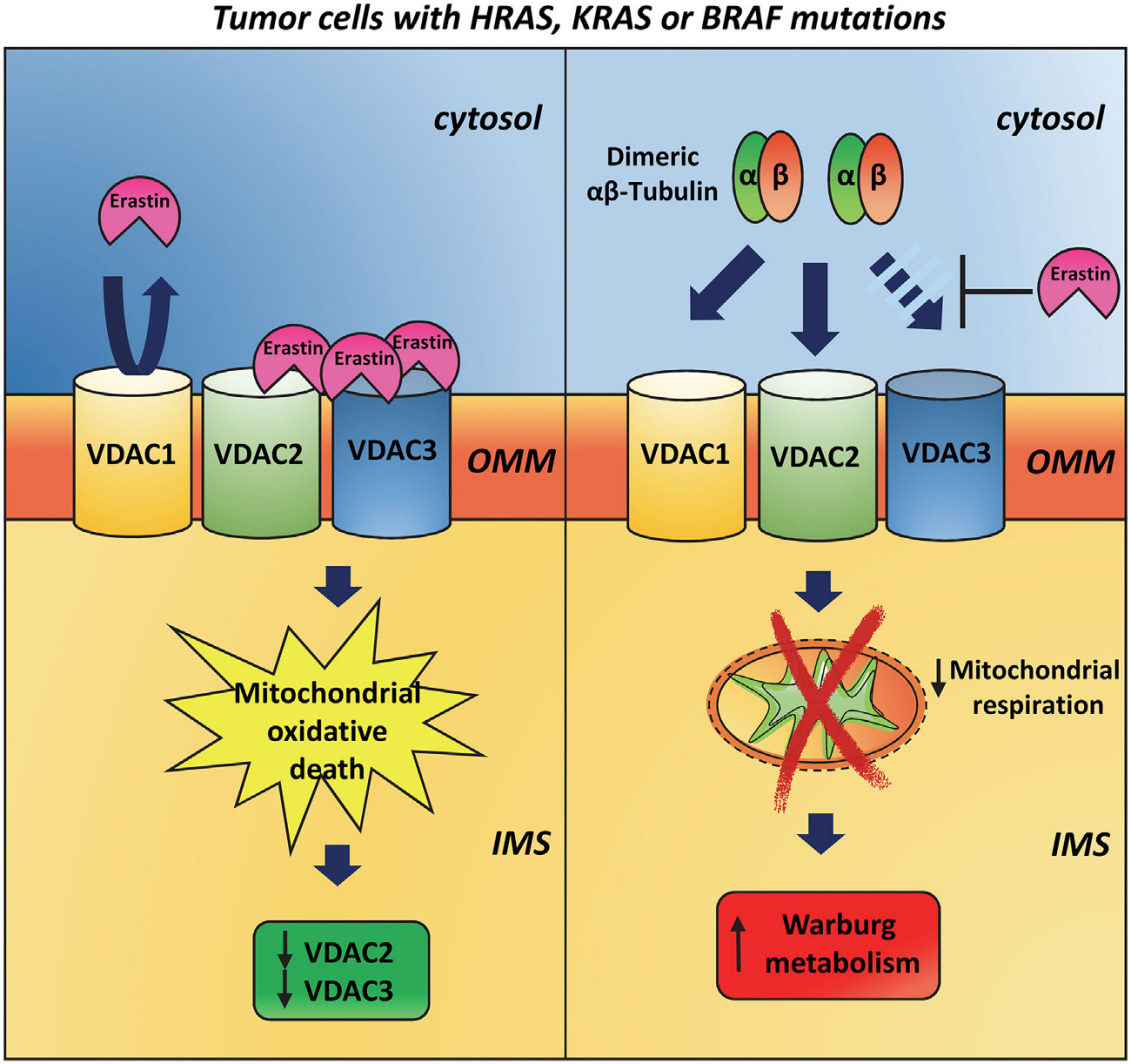
**Involvement of VDAC3 in cancer**. The anti-tumor agent erastin induces rapid, oxidative, non-apoptotic death in human tumor cells that have mutations in the oncogenes HRAS, KRAS, or BRAF. On the outer mitochondrial membrane, erastin binds VDAC2 and VDAC3 but is not able to interact with VDAC1. After erastin treatment, VDAC2 and VDAC3 expression is strongly reduced, while VDAC1 is still present at later time points (*left panel*). Dimeric αβ-tubulin is able to inhibit VDAC1 and VDAC2 conductance more than VDAC3 ones. This interaction suppresses mitochondrial respiration and contributes to the establishment of the Warburg metabolism in tumor cells. Erastin prevents and reverses tubulin-induced voltage-dependent anion selective channel blockage and promotes mitochondrial metabolism (*right panel*).

Evidence for a VDAC3 responsibility in specific mechanisms connected to cancer come from studies with erastin. The anti-tumor agent erastin induces oxidative, non-apoptotic death in human tumor cells with mutations in the oncogenes *HRAS, KRAS*, or *BRAF*. Using affinity-based target identification and MS/MS analysis, Yagoda et al. (82) identified VDAC2 and VDAC3 as the docking sites for erastin binding to mitochondria. In response to oncogenic *HRAS*, the total amount of VDAC protein is increased suggesting that erastin acts by a gain-of-function mechanism and that cells with more VDAC protein are more sensitive to erastin. Following erastin treatment, however, VDAC3 first and subsequently VDAC2 become undetectable. Hence, it is possible to hypothesize that a cellular response to erastin is the downregulation of VDAC2/3 after the generation of lethal oxidative species (Figure 3). On the contrary, VDAC1 is not altered after erastin exposure suggesting that the loss of VDAC2/3 is not simply caused by loss of mitochondria. The K.O. of VDAC3 significantly increases cell resistance to erastin and similar results are reported for VDAC2 K.O. In contrast, overexpression of VDAC3 alone does not increase sensitivity to erastin, suggesting that VDAC3, and partially VDAC2, is necessary but not sufficient to raise sensitivity to erastin, and that other downstream features of RAS–RAF–MEK signaling are necessary. In 2013, Maldonado et al. (83) discovered that erastin prevents and reverses tubulin-induced VDAC blockage to promote mitochondrial metabolism. As already reported, dimeric αβ-tubulin is able to block the conductance of VDAC and suppress mitochondrial respiration (84, 85). Single and double K.O. of VDAC isoforms in HepG2 cells showed that endogenous tubulin inhibits VDAC1 and VDAC2 conductance more than VDAC3 conductance (86). It has been speculated that the interaction between VDAC and tubulin may underlie modulation of energy metabolism in proliferating cells. During interphase, high free tubulin inhibits VDAC fluxes of ATP/ADP and other respiratory substrates across OMM, thereby suppressing mitochondrial respiration and promoting aerobic glycolysis in order to increase biomass formation. The decrease of free tubulin accompanying the cell division, instead, leads to the VDAC opening and the activation of oxidative phosphorylation that in turn generates the energy needed for chromosome movement and cytoplasmic division (86). In the light of the above results, the interaction between free tubulin and VDAC in cancer cells, which frequently show numerous alterations in the microtubule network (87), possibly contributes to the establishment of the Warburg metabolism (86).

## VDAC3 in Diseases Different from Cancer

Some reports in the literature show the involvement of VDAC3 in pathologies and diseases different from cancer. Chronic unpredictable stress (CUS), one of the most clinically relevant stress paradigms in rodents, mimics several behavioral characteristics of patients affected by depression, anxiety, and mood disorders. In a zebrafish model of CUS, Chakravarty et al. identified VDAC3 as a differentially regulated gene among other proteins involved in mitochondrial function (80). The authors hypothesized that the increased level of VDAC3 in stressed fish brain might increase the efficiency of bioenergetic metabolism and/or protection against ROS (80).

Alterations in the expression levels of VDAC3 have also been reported in the brain of mice infected by *Plasmodium berghei*, a well-studied mouse model of cerebral malaria (81). Cerebral malaria is the most severe neurological complication of infections by *Plasmodium falciparum*. This disease, mostly diffused in sub-Saharian Africa, has a high mortality and surviving patients generally manifest long-term neurocognitive impairment. The mechanisms underlying the brain injury are still unclear, and the upregulation of *vdac3* is accompanied by alterations of many other genes whose dysfunction is associated with neurological disorders (81).

## The Interactions between VDAC3 and Cytoskeletal Proteins are the Basis for Disorganization of Endocellular Functions?

The term “ciliopathies” refers to a series of genetic disorders associated with abnormal formation or function of cilia. The ciliopathies cause multisystem pathologies that can manifest with a plethora of features including retinal degeneration, cerebral anomalies, skeletal dysplasia, and fibrocystic diseases of the liver. It has been reported that VDAC3 is targeted to centrosome where it is mainly associated to the mother centriole/basal body (75). Here, it recruits Mps1, a protein kinase essential for the spindle assembly checkpoint and the modulation of centriole assembly in vertebrates. Depletion of VDAC3 cause defects in centriole assembly and in cell cycle enter. Further evidences revealed that VDAC3 and Mps1 cooperate to promote ciliary disassembly, while VDAC3 might have the additional function to inhibit cilia assembly in cycling cells (76). Interestingly, an earlier study demonstrated that, in VDAC3 K.O. mice, structural defects in the microtubular organization of the sperm tail cause markedly reduced sperm motility (36). As a consequence, male mice lacking VDAC3 are infertile. The molecular relationship between this defect and the VDAC deficiency is not known. It was found that VDAC3 is transcribed at specifically high levels in testis (33). Histochemical staining of bovine testis showed that VDAC1 is present in Sertoli cells and VDAC2 (but not VDAC1) in spermatocytes (88). By means of isoform-specific antibodies and biochemical evidence, it has been found that VDAC2 and VDAC3 are abundant in mature spermatozoa and appear to be associated with outer dense fibers of the sperm flagellum (89). Interestingly, also an association of VDAC isoforms with spermatozoa and ovarian tissue organization was found in *Drosophila melanogaster* (90, 91). In contrast, the work by Liu et al. assessed that no difference in VDAC3 expression levels exists between normozoospermic fertile donors and infertile patients with idiopathic asthenozoospermia; they instead found a high expression of VDAC2 (92).

It is important to note that human spermatozoa undergo extensive redox regulated signaling since nitric oxide (NO) is involved in sperm motility, capacitation, acrosome reaction, and enhancement of sperm binding to zona pellucida. NO induces S-nitrosylation, a post-translational protein modification that regulates cellular signaling. VDAC3 was identified as a target of S-nitrosylation in spermatozoa (93). Okazaki et al. found that S-nytrosilation activated VDAC3 channel activity by breaking S–S bonds (50).

## Implications of VDAC3 in Viral Infections

Many viruses encode proteins targeting to mitochondria. These proteins are able to modulate apoptosis in infected cells. In general, all the anti-apoptotic viral proteins contain mitochondrial targeting sequences that are responsible for inserting the protein in the OMM. In contrast, pro-apoptotic proteins like the HBx antigen from hepatitis B virus (HBV) contain amphipathic α-helices. HBV is a small hepatotropic DNA virus responsible for acute and chronic liver disease worldwide. The chronic viral infection constitutes a risk factor for hepatocellular carcinoma. It can indeed not only evolve to cirrhosis but also impact on cell cycle regulation and tumor suppressor genes alterations. It was reported that in HepG2 and HuH7, two hepatocarcinoma cells lines, HBx colocalizes with VDAC3 in the OMM (94, 95) and causes alterations in mitochondrial membrane potential (ΔΨm) leading to the activation of the transcription factors STAT-3 and NF-κB (96). The activation of these factors is sensitive to antioxidants and to the overexpression of Mn-superoxide dismutase II, suggesting a potential role of ROS in the pathogenesis of the disease. The release of cytochrome *c* in response to the HBx-induced mitochondrial membrane potential variation is instead a late event, indicating that during the stage of chronic HBV infection, HBx expression may sensitize apoptosis in infected hepatocytes, generating HBV pathogenesis and favoring propagation of the viral particles. Since Bcl-2 family proteins bind to VDAC to regulate the release of cytochrome *c*, it has been proposed that VDAC3–HBx interaction might induce conformational changes that abolish the modulation of channel activity by Bcl-2. VDAC3 seems to be involved also in HHV-8 infection, an oncogenic human herpes virus that has been identified in all types of Kaposi’s sarcoma. Wang et al. (97) reported indeed that HHV-8 K7 protein binds to mitochondrial VDAC3.

## Conclusion

In mammals, VDAC3 has been demonstrated to be subjected to over-oxidation of its exposed cysteines. The oxidative modifications, likely due to the concentration of ROS in the IMS, let us propose that it can be candidate to participate in the molecular mechanisms of identification of damaged mitochondria. Damaged mitochondria characterize the mitochondrial dysfunction, a state of the organelle where the oxidative phosphorylation is not able to produce enough ATP and ROS accumulation is a hallmark. This state must be signaled as soon as possible, to remove the damaged organelle. The VDAC3 cysteine oxidations, which were experimentally proved (41), produce changes of the protein electrostatic map, and consequently changes in its presentation to the other compartments. The peculiar modifications of cysteines thus make VDAC3 a potential counter of ROS load in the IMS. The quest for a messenger able to specifically interact with the oxidized VDAC3 and transfer the message to proper responding apparatus will be the next step of this story.

## Author Contributions

AM and FG contributed to the bibliographic survey; SR and VP conceived the review and wrote the manuscript.

## Conflict of Interest Statement

The authors declare that the research was conducted in the absence of any commercial or financial relationships that could be construed as a potential conflict of interest.
